# Undergraduate-level teaching and learning approaches for interprofessional education in the health professions: a systematic review

**DOI:** 10.1186/s12909-021-03073-0

**Published:** 2022-01-03

**Authors:** Marwh Gassim Aldriwesh, Sarah Mohammed Alyousif, Nouf Sulaiman Alharbi

**Affiliations:** 1 Department of Clinical Laboratory Sciences, College of Applied Medical Sciences, King Saud bin Abdulaziz University for Health Sciences, Riyadh, Saudi Arabia; 2 King Abdullah International Medical Research Center, Riyadh, Saudi Arabia; 3 Ministry of the National Guard - Health Affairs, Riyadh, Saudi Arabia; 4 Department of Medical Education, College of Medicine, King Saud bin Abdulaziz University for Health Sciences, Riyadh, Saudi Arabia

**Keywords:** Interprofessional education, Multiprofessional education, Pre-licensure, Undergraduate, Teaching approaches, Learning approaches, Health professions

## Abstract

**Background:**

Although most systematic reviews of interprofessional education (IPE) evaluated the impact of IPE on the students’ acquisition of knowledge in relation to other professions, the development of teamwork skills, and the changes in collaborative behaviour, the processes involved in IPE (i.e., approaches to teaching and learning) are under-researched. The purpose of the study was to conduct a systematic review to establish how IPE has been implemented in university-based undergraduate curricula, focusing on the teaching and learning approaches.

**Methods:**

The systematic review was performed in 2020 with three databases: PubMed, Science Direct, and the Cochrane Library. Titles and abstracts were included based on pre-identified eligibility criteria. We used the article entitled ‘Systematic reviews in medical education: a practical approach: AMEE guide 94’ as the basis to establish the aim and methods of the current systematic review from 2010 to 2019.

**Results:**

We found 16 articles that met the inclusion criteria and reported the implementation process of IPE in universities from Western, Asian, and African countries. A combination of at least two teaching and learning approaches was used to deliver IPE. The findings indicated that of all the teaching and learning approaches, simulation-based education, e-learning, and problem-based learning were the most prevalent approaches used to deliver IPE. This systematic review also revealed a lack of IPE programmes in the Middle East region.

**Conclusions:**

The evidence synthesised in the current systematic review could support IPE curriculum planners and educators when planning an IPE programme. More global IPE initiatives are required to meet the global health workforce needs. Further studies are required to identify the effectiveness of the different teaching and learning approaches in the development of IPE competencies.

**Supplementary Information:**

The online version contains supplementary material available at 10.1186/s12909-021-03073-0.

## Background

The diagnosis, treatment, management and prevention of chronic and acute diseases require the collaborative efforts of various healthcare workers. Therefore, undergraduate teaching institutions specially focussed on healthcare education have the responsibility to teach and train students to ensure their ability to adapt to challenges and respond adequately to patients’ healthcare needs [1]. In most parts of the world, educational activities for every profession at the undergraduate level are performed independently from one another (i.e., each profession plans and conducts its own teaching). As a result, graduates have limited knowledge about other professions, limited communication, collaboration, and teamwork skills and must wait until they enter an actual work environment before they can interact with other professions. To overcome this limitation, the World Health Organization (WHO) formulated a study group to develop and globally implement a universal plan for interprofessional education (IPE) in 1988 [2, 3]. The WHO describes IPE as occasions when two or more candidates from different professions learn from, with and about each other to develop effective collaboration and enhance healthcare outcomes [1, 2]. IPE is designed to improve interaction, communication and teamwork skills between different healthcare professions [4]. It is necessary for different healthcare professionals to learn about the knowledge, skills, and expertise of each person on the team in order for the team to function well. Thus, IPE should be established in an ethical and transparent learning environment that is safe and where the needs of all healthcare professionals are given equal consideration and their diversity is recognised [5]. As reported by the WHO, IPE does not substitute the undergraduate curriculum for a single profession but complements it [2].

The extant literature identified four interprofessional competency frameworks: the interprofessional capability framework that originated in the United Kingdom in 2004, the national interprofessional competency framework developed in Canada in 2010, the core competencies for interprofessional collaborative practice established in the United States in 2011 and the interprofessional capability framework produced by Curtin University in Australia in 2011 [6]. These frameworks have shared domains associated with interprofessional communication, role clarification and team functioning [6]. Thus, these competency domains are crucial as a platform when planning IPE-based curricula and for guiding medical educators to identify the appropriate teaching and learning approaches and assessment tools to achieve these competencies [7]. The IPE activities are based on adult learning principles [2] and are commonly associated with teaching and learning approaches that improve the competencies highlighted by the four frameworks mentioned above. IPE activities have their roots in transformative learning theory, as team members adjust their existing beliefs about their professions and other healthcare professions based on new IPE experiences [8]. Regardless of the similarities in the competency domains of interprofessional frameworks, the frameworks focus on different aspects of outcomes and processes at one of two levels: individual level or team level [6]. The two interprofessional capability frameworks, one originating from the United Kingdom and the other from Australia, and the core competencies for interprofessional collaborative practice framework are focused on the outcomes of IPE at the individual level [6]. On the other hand, the fourth framework, the national interprofessional competency framework, focuses on the processes used by IPE teams [6].

In addition to the interprofessional competency frameworks, the University of British Columbia (UBC) developed a model for IPE that provides a framework for conceptualising IPE learning processes [9]. The UBC IPE model has three phases: exposure, immersion and mastery [9]. In the introductory phase (exposure), learners are expected to have a clear and deep understanding of their profession while also being aware of the roles of other professions [9]. In the second phase, learners gain core knowledge and skills in their own professions [9]. In addition, learners are exposed to other professions in their clinical placements [9]. Meanwhile, the advanced phase is for practitioners who ‘master’ interprofessional concepts in their daily professional practice and have a comprehensive understanding of their own as well as other professions [9, 10].

Most systematic reviews related to IPE evaluated the impact of IPE on the students’ acquisition of knowledge in relation to other professions and the development of collaborative and teamwork skills and changes in collaborative behaviour [11–17]. However, the processes involved in IPE (i.e., approaches to teaching and learning) are under-researched. In a systematic review reporting several teaching and learning approaches related to the implementation of IPE in universities across the globe, problem-based learning (PBL), case-based learning (CBL) and team-based learning (TBL) were identified as the most frequent teaching and learning approaches in IPE [18]. PBL is a learning approach in which learning is acquired by solving or understanding a clinical problem [19]. A classical PBL tutorial includes a group of students (usually eight to 10) who work together in sequential steps to eventually solve a clinical problem, and a tutor to guide the students and support their learning [20]. In PBL, each participant has a clear role to play: a scribe who is responsible for writing notes discussed by group; a tutor who facilitates the PBL session; a chair who manages the group through the learning process; and group members who follow the process of PBL in sequence. The roles are rotated between students for each clinical scenario [20]. Group learning through PBL is proposed to be mostly a social process where members of a learning community collaborate to discuss and negotiate a clinical problem [21]. Members of a PBL tutorial meet and discuss their notes after individual learning, which enables the students to acquire knowledge and develop specific skills and attitudes related to communication, teamwork, collaboration, respect for peers’ perspectives and information sharing [20, 21]. Furthermore, the Linköping model of IPE at the undergraduate level, which was first implemented in 1986 and included six educational healthcare programmes (laboratory, physiotherapy, occupational therapy, nursing, medicine and social services), utilised PBL as a main learning approach [22, 23]. After 15 semesters with more that 4000 students, the outcomes were extremely positive in aspects related to knowledge, skills and attitude, which could improve teamwork and cooperation, thereby improving the quality of patient care in real-life clinical practice [22, 23]. The study author concluded that IPE in combination with PBL is feasible and recommended that it could be implemented in undergraduate curricula [22, 23].

CBL and TBL are two alternative learning approaches to PBL. They are based on the same learning theories (e.g. experiential learning), share common objectives (e.g. promoting self-directed learning) and all rely on collaboration and teamwork for their success [24, 25]. However, CBL requires advance preparation by the learners for the purpose of problem-solving, whereas TBL is not restricted to clinical cases, as it can utilise a wide variety of clinical problems. Additionally, TBL puts an emphasis on students mastering the course content before problem-solving [24, 25]. Just like PBL, both case- CBL and TBL are recommended to achieve IPE-related learning objectives, such as understanding the role of all members in the healthcare team and acknowledging the value of collaboration with other healthcare professions [26, 27]. In addition to PBL, CBL and TBL, simulation-based education was highlighted as an effective learning approach for achieving IPE competencies at certain universities [18]. Simulation-based education provides a secure learning environment for students from different professions to learn with, about and from each other in settings that mimic real-life clinical experiences with proper guidance [28, 29]. The concept underpinning the positive impact of simulation on the communication and collaboration skills of learners in IPE can be illustrated as a cyclical process, based on Kolb’s experiential learning theory (ELT) [28, 29]. Like simulation, e-learning has been used as a learning approach for IPE in the pre-licensure stages of education [30]. The term e-learning refers to the use of electronic platform(s) to deliver learning [31]. The e-learning approach motivates self-directed learning, whether it is synchronous (i.e. learner and instructors are engaged in learning activities at the same time) or asynchronous (i.e. learners are engaged in learning on their own time based on a pre-set learning path) [30]. E-learning has many positive aspects that makes it suitable for the implementation of IPE [32–34]. For instance, it provides a more flexible environment in terms of time, scheduling, and geography. It is also an effective learning approach for achieving the IPE competencies and promoting a collaborative learning environment [35, 36]. The electronic interaction of learners facilitates the understanding of one’s role and the roles of others. This interaction might be between the learners themselves, between learner and learning materials, or between learner and mentor [34]. Utilisation of an e-learning management system, such as WebCT or Blackboard, has the ability to foster effective communication between learners [34].

Previous work by Khalili and collaborators [37, 38] revealed the importance of interprofessional socialisation in professional educational curricula, especially in the early stages of professional education. Khalili and his colleagues developed the interprofessional socialisation framework, which proposes the progress of interprofessional socialisation through three stages [37, 38]. In the first stage, learners’ uniprofessional identities are challenged by placing them in situations where they have to address misconceptions about their roles and deal with aggressive attitudes about their profession from professionals in other fields. These challenges motivate the learners to find solutions and be open-minded about other professions by learning about, with, and from them [37, 38]. In other words, this stage facilitates learners breaking down the barriers that can hinder cooperation and collaboration. In the second stage, the IPE environment starts to flourish, as learners start developing IPE competencies, such as learning about the roles of other professions, through working on a simulated patient in interprofessional teams [37, 38]. The dual identity fully develops in the last stage, as this is where learners advance their understanding of their role in their own profession and develop a sense of belonging in the interprofessional community [37, 38]. Regardless of the efforts spent in exploring IPE, there is still a lack of evidence on the implementation of IPE in undergraduate curricula. The current study aimed to systematically review the literature on IPE processes and to highlight these sources as useable references for decision-makers involved in IPE initiatives. The following research question guided the review: ‘What are the IPE teaching and learning approaches in the undergraduate curricula for the healthcare professions?’

## Methods

We initially conducted a non-systematic search within relevant journals for IPE related publications (i.e., Journal of Interprofessional Care, Journal of Interprofessional Education and Practice, Medical Teacher, Clinical Teacher, and Education for Health) to identify existing systematic reviews related to teaching and learning approaches of IPE. However, the available systematic reviews related to IPE were principally focused on the effectiveness and outcomes of IPE and paid little attention to the teaching and learning approaches. The current review was developed using the systematic reviews in medical education: a practical approach: AMEE guide 94 [39].

The study team consisted of a researcher with experience in systematic review methods, medical educators with a strong background in IPE, a medical librarian familiar with searching different databases and who provided guidance in the documentation process, and a data manager capable of managing a large quantity of abstracts, tracking the status of each abstract (included or excluded) and updating the Preferred Reporting Items for Systematic Reviews and Meta-Analyses (PRISMA) flow diagram. Afterwards, the team wrote a detailed and clear plan for the systematic review protocol. The protocol was used to guide the review process and track the documents to verify the quality of the work performed. To formulate the current systematic review’s question, the participants, educational aspects and outcomes (PEO) model [40] was used. This process involved the following factors: (1) participants: undergraduate students of healthcare professions; (2) educational aspect: IPE; (3) outcome: teaching and learning approaches of IPE.

Searches were conducted in the following electronic databases: PubMed, Science Direct and the Cochrane Controlled Trial Register. Specifically, full-text articles published in peer-reviewed journals in English from 1 January 2010 to 31 December 2019 were searched by combining the following terms: ‘interprofessional education’ OR ‘interprofessional learning’ OR ‘multiprofessional education’ OR ‘multiprofessional learning’ AND ‘undergraduate’ AND ‘prelicensure’ AND ‘prequalification’ (Additional file 1). We included articles that reported the teaching and learning approaches of the IPE program in the university-based curricula for undergraduate healthcare professions. The systematic review focused on the undergraduate healthcare professions students engaged in an IPE learning environment regardless of gender, age, or geographical location. The healthcare professions included, but were not limited to, medicine, dentistry, nursing, pharmacy, and allied health, which includes nutrition, occupational therapy, physical therapy, audiology, speech pathology, respiratory therapy, and radiology. Reviews, perspective articles, conference proceedings, graduate theses and commentaries were excluded from the current review.

To minimise random error and bias, at least two of the study team worked on the inclusion and exclusion processes with the articles. Two members screened the titles, abstracts, and keywords. An article was excluded if both members agreed. In situations where insufficient details were provided, the article was moved to the next search stage. During this stage, the full text was read by the two members to make a final decision about the inclusion or exclusion of the article. Any disagreement between the two members was resolved by a third team member to achieve consensus. During the stages of article selection, the justifications for an exclusion were precisely documented. The PRISMA flow diagram (available at http://www.prisma-statement.org/) was used to illustrate the process of searching and selecting the primary articles included in the review. In this phase, all the articles that met the inclusion criteria were included regardless of their quality. The quality assessments of the articles were performed in subsequent stages.

The extracted data and quality assessments were performed by two independent reviewers. Any conflicts raised between the reviewers were resolved by a third reviewer until consensus was reached [41]. Due to the nature of the current review, descriptive data were extracted using a data extraction tool that was specifically generated to address the aim of this review (Additional file 2). The tool contained information obtained from each article, such as the article title, author(s), and publication year. It also included the institutions and countries that implemented IPE programs as part of their undergraduate curricula, settings, contexts, and the teaching and learning approaches for IPE. Details related to the placement of IPE within curricula were also gathered. In addition, the methods and reported findings used to evaluate the IPE programs were collected.

For the quality assessment, key details were extracted from each article and assessed using a quality checklist appraisal form. The Mays et al.’s quality checklist for mixed-methodology case studies and other in-depth complex designs, developed in 2001 was adapted and utilized to assess quality of the articles [42] (Additional files 3 and 4). The checklist included criteria related to the transparency of ethical issues, the intended aims of IPE programs, IPE implementation processes, participating healthcare professions, contexts, and funding sources. It also contained assessment criteria for evaluating the IPE programs, such as the clarity of the study design, sampling, data collection, data analysis, results, and conclusion. Furthermore, the outcomes of the IPE programmes implemented in the included studies were assessed using the six-level modified Kirkpatrick’s framework [43, 44]. The different outcome levels of this framework capture learners’ reactions, changes in attitudes, knowledge or skills and changes in organisational practice and patients’ outcomes (Additional file 5) [43, 44].

## Results

### Search results

**Fig. 1:**
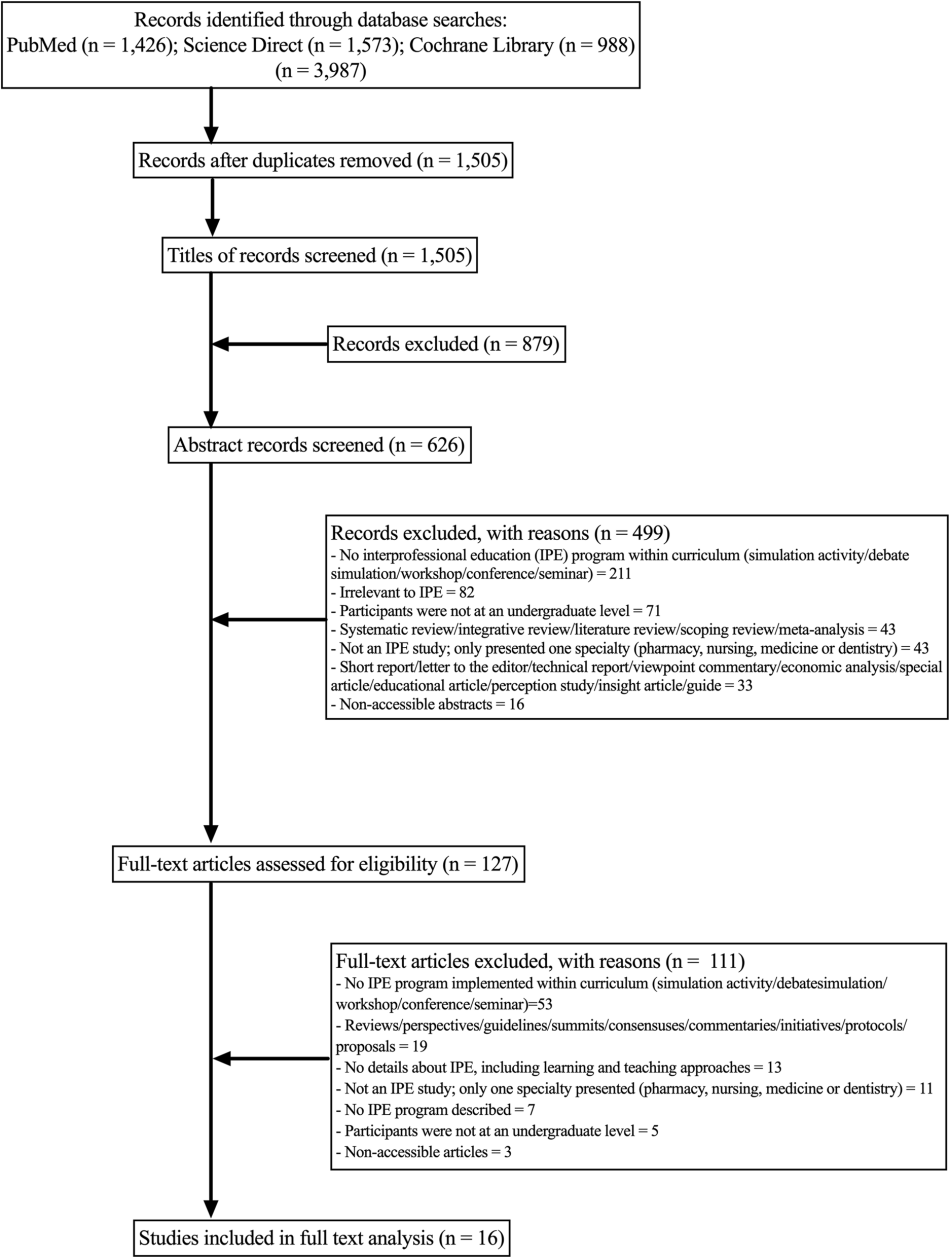
Flow chart depicts process performed to retrieve articles for inclusion in systematic review

Following an extensive literature search, a total of 3987 articles were identified from the databases (1426 from PubMed, 1573 from Science Direct, and 988 from the Cochrane Library) (Fig. 1). Of these, 2482 duplicates were identified, resulting in 1505 articles to be screened by title and abstract. From these, 127 articles were assessed for eligibility to be included in the current review. After retrieving the full-text review for intensive evaluation, 111 articles were excluded due to the absence of some details required to be eligible (e.g., no IPE program implemented within curriculum, no details about IPE, including teaching and learning approaches, not an IPE study, only one speciality presented, participants were not at an undergraduate level). As a result, 16 studies met the inclusion criteria and were eligible to be included in the current review [1, 30, 45–58].

### Quality assessment

The studies included (*N* = 16) varied in quality (Table 1). Most studies focussed on IPE program aims, design, origin, nature, context, implementation mechanism, conclusion, and ethical considerations. The quality criteria related to sampling, data collection, data analysis, and results were not applicable in more than half of the studies [45–49, 54–57]. Ten of the studies did not mention whether the IPE program was supported financially [30, 47–49, 51, 54–58]. Finally, in 14 studies, the authors did not state whether any conflicts of interest existed [30, 45–51, 53–57].

**Table 1:** Results of the critical appraisal of the articles included in the systematic review

Citation	Did the study/project address a specific question/aim?	Was the study design explained?	Did the paper clarify if the study/project was funded? If yes, by whom?	Was the origin of the study/project stated?	Was the nature/background of the innovation illustrated?	Was the context of the study/project sufficiently described?	Were the participants in the study/project elucidated?	Was the implementation process explained?	Did the authors include sufficient cases / settings / observations so that conceptual rather than statistical generalisations could be made?	Was the data collection process systematic, thorough and auditable?	Were data analysed systematically and rigorously? Were sufficient data presented?	Were the main results stated with enough details?	Did the authors draw a clear link between data and explanation (theory)?	Were the authors’ positions and roles clearly explained and the resulting biases considered?	Was the declaration of interest stated?
Van Lierop et al. (2019) [51]	Yes	Yes	No	Yes	Yes	Yes	Yes	Yes	No	No	Unclear	No	Yes	No	Yes
Rodríguez et al. (2019) [52]	Yes	Yes	Yes	Yes	Yes	Yes	Yes	Yes	Yes	Yes	Yes	Yes	Yes	Yes	Yes
Van Gessel et al. (2018) [1]	Yes	Yes	Unclear	Yes	Yes	Yes	Yes	Yes	Yes	Yes	Unclear	Yes	Yes	Yes	Yes
Imafuku et al. (2018) [53]	Yes	Yes	Yes	Yes	Yes	Yes	Yes	Unclear	Yes	Yes	Yes	Yes	Yes	No	Yes
Milot et al. (2015) [54]	Yes	No	No	Yes	Yes	Yes	Yes	Yes	NA^a^	NA	NA	NA	No	No	Yes
Sanborn H. (2016) [30]	Yes	Unclear	No	Yes	Yes	Yes	Yes	Yes	Unclear	Unclear	Unclear	Unclear	No	No	Yes
Horsley et al. (2016) [55]	Yes	Yes	No	Yes	Yes	Yes	Yes	Yes	NA	NA	NA	NA	Yes	No	No
Meche et al. (2015) [56]	Yes	Yes	No	Yes	Yes	Yes	Yes	Unclear	NA	NA	NA	NA	No	No	Yes
Waggie M. and Laattoe N. (2014) [57]	Yes	Yes	No	Yes	Yes	Yes	Yes	Unclear	NA	NA	NA	NA	No	No	Yes
Hinderer K. and Joyner R. (2014) [58]	Yes	Yes	No	Yes	Yes	Yes	Yes	Yes	Yes	Yes	Yes	Yes	Yes	No	No
Vanier et al. (2013) [45]	Yes	Yes	Yes	Yes	Yes	Yes	Yes	Yes	NA	NA	NA	NA	Yes	No	No
Holland et al. (2013) [46]	Yes	Yes	Yes	Yes	Yes	Yes	Yes	Yes	NA	NA	NA	NA	Yes	No	Yes
Doucet et al. (2013) [47]	Yes	Yes	No	No	Unclear	Yes	Yes	Yes	NA	NA	NA	NA	Yes	No	Yes
Pardue K. (2013) [48]	Yes	Yes	No	Yes	Yes	Yes	Yes	Yes	NA	NA	NA	NA	Yes	No	Yes
Olenick et al. (2011) [49]	Yes	Yes	No	Yes	Yes	Yes	Yes	Yes	NA	NA	NA	NA	Yes	No	Yes
Bilodeau et al. (2010) [50]	Unclear	Yes	Yes	Yes	Unclear	Yes	Yes	Yes	Unclear	Unclear	Yes	Yes	Yes	No	Yes

^a^ Not applicable

As shown in Table 2, the evaluation of IPE programme outcomes based on the modified Kirkpatrick’s framework revealed changes in IPE participants’ knowledge or skills (Level 2b) in 50% (*n* = 8) of the included studies [45–47, 52, 53, 55, 56, 58]. Six studies (37.5%) reported changes in pre-licensure students’ attitude (Level 2a) toward IPE [1, 45–47, 51, 58], whereases seven studies (43.75%) demonstrated a change in organisational practice (Level 4a) after the implementation of IPE [30, 46, 48–50, 57, 58]. A limited number of studies indicated a change in students’ participation reaction (Level 1) [58] and in clinical outcomes (Level 4b) [54] after implementation of the IPE programme (Table 2).

**Table 2:** Evaluation results (based on the modified Kirkpatrick’s framework) of the outcomes of the interprofessional education (IPE) programmes implemented in the articles included in the systematic review

Citation	Classification of IPE outcomes	Description
Van Lierop et al. (2019) [51]	Level 2a	**Change in attitude:** Positive experience, opportunity to learn IPE, collective responsibility for patient care and better understanding of context.
Rodríguez et al. (2019) [52]	Level 2b	**Change in knowledge or skills:** IPE promoted the development of higher order thinking skills such as research.
Van Gessel et al. (2018) [1]	Level 2a	**Change in attitude:** Positive attitude toward the implementation of IPE training.
Imafuku et al. (2018) [53]	Level 2b	**Change in knowledge or skills:** Facilitated understanding of communication, teamwork, and identity formation as health professional.
Milot et al. (2015) [54]	Level 4b	**Change in clinical outcomes:** Positive feedback from clinical setting.
Sanborn H. (2016) [30]	Level 4a	**Change in organizational practice:** New IPE learning objectives and learning activities integrated through 10 courses.
Horsley et al. (2016) [55]	Level 2b	**Change in knowledge or skills:** Establish and improve team performance.
Meche et al. (2015) [56]	Level 2b	**Change in knowledge or skills:** IPE skills development (e.g., communication and collaboration).
Waggie M. and Laattoe N. (2014) [57]	Level 4a	**Change in organizational practice:** Assist in the process of developing IPE curricula.
Hinderer K. and Joyner R. (2014) [58]	Level 1, Level 2a, Level 2b and Level 4a	**Participation reaction:** Exposure to faculty from another profession and content usefulness. **Change in attitude:** Attitude about IPE collaboration **Change in knowledge or skills:** Seeking guidance from each other in IPE. **Change in organizational practice:** Elective undergraduate interprofessional critical care course.
Vanier et al. (2013) [45]	Level 2a and Level 2b	**Change in attitude:** Confidence level, learning and course appreciation. **Change in knowledge, or skills:** Informal comments received from clinical settings.
Holland et al. (2013) [46]	Level 2a, Level 2b and Level 4a	**Change in attitude:** Enhance understanding of each other’s knowledge and skills. **Change in knowledge, or skills:** Evaluate evidence underpinning IPE care, integrate clinical knowledge, decision making, and reflective practice in IPE care. **Change in organizational practice:** Increases in the number of faculty staff competent in IPE pedagogy.
Doucet et al. (2013) [47]	Level 2a and Level 2b	**Change in attitude:** Positive experience with IPE. **Change in knowledge, or skills:** Enhancement in communication and collaboration skills.
Pardue K. (2013) [48]	Level 4a	**Change in organizational practice:** Introduction of innovative undergraduate IPE curriculum.
Olenick et al. (2011) [49]	Level 4a	**Change in organizational practice:** Regional IPE model integrated into the curricula of 14 different health professional schools.
Bilodeau et al. (2010) [50]	Level 4a	**Change in organizational practice:** Development of integrated IPE training program.

### Findings of included studies

To ensure the literature reflected the current knowledge related to IPE, the studies included were limited to a publication date from 2010 to 2019. Additional file 6 summarized the characteristics of studies included reporting IPE program implementation in undergraduate curricula. The studies included represent countries from Western, Asian, and African populations. Of the 16 studies, five were from the USA [30, 48, 49, 55, 58], four from Canada [45, 47, 50, 54], two from Switzerland [1, 56], one from Spain [52], one from the UK [46], one from the Netherlands [51], one from Japan [53] and one from South Africa [57]. According to the synthesized evidence, a combination of at least two teaching and learning approaches was used to deliver IPE, as shown in Additional file 6. The findings showed that of all the teaching and learning approaches, simulation-based education [1, 46, 48–50, 55, 56, 58], e-learning [30, 46, 47, 49, 50, 54, 56, 57] and PBL [48, 49, 51, 53, 57, 58] were the most prevalent approaches implemented to deliver IPE. Manikin-based simulation was used in three studies [46, 55, 58], standardized patient simulation was utilized in two studies [1, 49], skills training simulation was applied in two studies [48, 56], and one study did not indicate type of simulation used [50]. Other teaching and learning approaches included in-practice teaching [46, 53, 58], didactic input [50, 53, 57], competency-based learning [30, 45], self-directed learning [30, 46], blended learning [46, 54], evidence-based practice [48, 49], and experiential learning [30, 53]. The least frequent teaching and learning approaches used to deliver IPE were open inquiry-based learning [52], team-based learning [47], and an interprofessional congress [1] (Table 3).

**Table 3:** Teaching and learning approaches implemented in the interprofessional education of the undergraduate curricula

Learning approach	Interprofessional education studies, n (%)	Tools used
Simulation-based learning	8 (50)	• Simulated patients • Simulation workshops
E-learning^a^	8 (50)	• Virtual games • Discussion boards • Live web-based seminars • Web-based discussion forums • Virtual environment interactive exercises • Asynchronous discussions facilitated by a website
Problem-based learning	8 (50)	• Case studies • Case-based interprofessional sessions
In-practice teaching	3 (18)	• Early exposure • Clinical observation
Didactic input	3 (18)	• Lectures and interactive lectures-based teaching
Competency-based learning	2 (12)	• Unclear
Self-directed learning	2 (12)	• Assignments • Written papers • Individual reading
Blended learning	2 (12)	• E-learning (synchronous) • Face-to-face activities
Evidence-based practice	2 (12)	• Unclear
Experiential learning	2 (12)	• Laboratory work
Open inquiry-based learning	1 (6)	• Unclear
Team-based learning	1 (6)	• Unclear
Interprofessional congress	1 (6)	• Workshops • Plenary sessions

^a^E-learning was synchronous in four studies, asynchronous in one study, a combination of both synchronous and asynchronous in one study and unclear whether synchronous or asynchronous in two studies

Based on the studies reviewed (*N* = 16), nursing, medicine, and physiotherapy were the healthcare professions that most frequently participated in IPE programs, at 87.5% (*n* = 14), 62.5% (*n* = 10) and 62.5% (*n* = 10), respectively. Other healthcare professions that participated in IPE programs included nutrition, pharmacology, medical radiology, and respiratory therapy. As depicted in Additional file 6, the university-based IPE programs were implemented in 14 studies (87.5%) [1, 30, 45, 47, 48, 50–58]. Few studies based their IPE programs in university and hospital [50, 53] or in university and community settings [57]. The IPE program was integrated in the existing undergraduate curricula in seven studies [1, 30, 46, 49, 50, 53, 54]. However, the mechanism of IPE involvement in undergraduate curricula was unclear in six studies [45, 47, 48, 56–58] (Additional file 6).

**Fig. 2:**
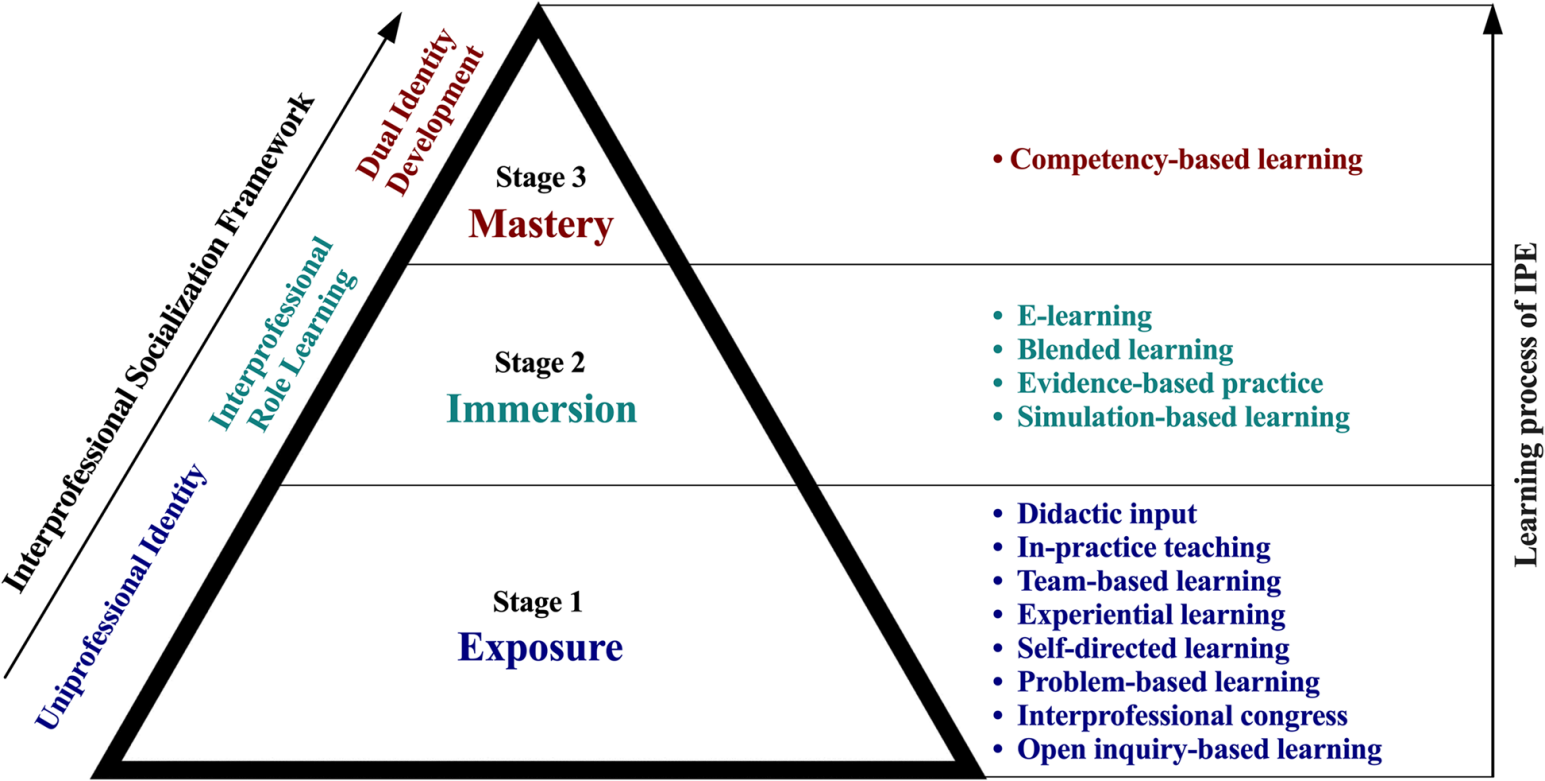
Learning and teaching approaches of interprofessional education (IPE) based on the University of British Colombia’s model [9] of IPE and interprofessional socialization framework [38]

To analyse the results further, the UBC model [9] and Khalili’s socialization framework of IPE [38] were utilised, as shown in Fig. 2. The IPE learning and teaching approaches of undergraduate curricula in the included studies (*N* = 16) were categorised into stages, according to both the UBC model of learning processes and the interprofessional socialisation framework. These stages are: (1) awareness and exposure (uniprofessional identity); (2) immersion and application (interprofessional role learning); and (3) mastery and competence (dual-identity development). According to the synthesised evidence, most learning and teaching approaches belonged to the awareness and exposure category, where the uniprofessional identity is developed (*n* = 13, 81.25%) [1, 30, 45–53, 57, 58]. This was followed by the immersion and application stage of learning, where IPE evolves and collaboration with other professions becomes more frequent (*n* = 9, 56.25%) [1, 46, 48–50, 54–56, 58]. The mastery stage of learning, where IPE thrives and the dual identity is formed, was the least implemented (*n* = 2, 12.5%) [30, 45]. This is represented by competency-based learning (Fig. 2).

## Discussion

Selecting appropriate teaching and learning approaches to deliver IPE is a key factor in ensuring the achievement of IPE competencies related to communication, teams and teamwork and roles and responsibilities. The present systematic review aimed to synthesise the available evidence related to IPE teaching and learning approaches in the undergraduate curricula of healthcare-related fields. Based on the evidence, a combination of at least two teaching and learning approaches was used. Simulation-based learning, e-learning and PBL were the most frequently reported teaching and learning approaches for IPE.

Based on the literature, simulation can be used as a learning approach in IPE to promote effective communication and collaboration in healthcare profession students [28]. According to ELT, learners move through sequential phases during simulation-based IPE: (1) concrete experience through participating in the simulation activity itself; (2) reflective observation via debriefing and reflection; (3) abstract conceptualisation considering the relevance of the IPE activity; and (4) active experimentation by testing what was learned [28]. The advantage of simulation-based IPE is that it is suitable for different types of learners, for instance, divergent learners, who achieve the best learning outcomes through concrete experience, reflective observation and involvement in teams and idea development; assimilating learners, who achieve the best learning outcomes through reflective observation and abstract conceptualisation; converging learners, who achieve the best learning experience through abstract conceptualisation and active experimentation; and finally, accommodating learners, who prefer concrete experience and active experimentation [28, 29]. Simulation could eventually complement various types of learners and provide an effective means to acquire IPE competencies. Various e-learning tools were used in the studies included in this review, which were effective for the healthcare students, such as live web-based seminars, virtual environment interactive exercises and web-based discussion forums [30, 54, 56, 57]. A recent study published by Edelbring and colleagues in 2021 examined the feasibility of implementing a blended online virtual patient (VP) approach for nursing and medical students from two independent courses [59]. Most of the nursing and medical students (173 out of 201, 86%) selected the online platform to meet and learn due to its flexibility in terms of time and space. Moreover, the study participants reported that the use of flexible online activities based on VPs was useful for improving their understanding and awareness of other profession’s roles and responsibilities, which is one of the major IPE competencies [59]. It also provided a comprehensive overview of a patient’s clinical case by combining medical and nursing students’ perspectives. The study authors concluded that the blended online VP approach is effective for IPE delivery [59]. Furthermore, previous studies demonstrated that e-learning is both time- and cost-effective, allowing for socialisation and sharing of resources and experiences between different healthcare professions [32–34]. Despite the advantages of e-learning in IPE, it works best when paired with other methods. The integration of e-learning with other face-to-face learning activities that provide students with the opportunity to practice what they have learned virtually is essential [34]. To justify the use of PBL in IPE delivery, it is important to describe the features of the IPE experience quality [60]: (1) learning is shared in the different professions [60, 61]; (2) learners compare and contrast their roles and responsibilities [60, 61]; (3) learning is an interactive process [60, 61]; (4) reflection is a key element in the learning process [60, 61]; (5) learning activities should involve experiential learning [60, 61]; (6) planning includes team members from multiple professions [60, 61]; (7) collaboration is an essential learning outcome [60, 61]; and (8) learning activities are designed to challenge stereotypes [60, 61]. There is an alignment between the IPE quality features and the PBL principles. The characteristics of interactive, reflective and experiential learning are the cornerstones of uniprofessional PBL [61]. Overall, the implementation of PBL in IPE contributes fundamentally to achieving IPE competencies related to roles and responsibilities, interprofessional communication and team functioning.

The results of the present review revealed that PBL is more frequently used with undergraduate IPE activities, while CBL and TBL were rarely utilised. Regardless of how frequently each approach was used, the three share common challenges in the context of delivering IPE activity across different groups of healthcare professional learners [24, 61]. The first challenge is related to the logistics. For example, setting timetables for an IPE activity that accommodate more than one curriculum for more than one healthcare profession is not easy. Another logistical challenge associated with IPE implementation is the need for more facilitators who are well trained to lead PBL, CBL or TBL approaches for an IPE activity. The third challenge arises from the differences in the learning needs of each individual and profession [24, 61, 62]. This difference requires careful consideration of the application of the learning approach used to ensure those needs are addressed and that the educational objectives are met. Assessment presents another challenge for PBL, CBL and TBL in IPE, as it is difficult to ensure the validity of the results due to the wide range of factors playing a role in these learning activities [24, 61, 62]. Although PBL shares these challenges with CBL and TBL, it is more frequently used because it focusses on teaching learners the process of problem solving by promoting independent learning and teamwork [61]. This focus is crucial when dealing with undergraduate students, who are still learning the basis of scientific inquiry in their profession. Conversely, CBL and TBL focus on advanced problem-solving skills in a way that puts more responsibility on the learner while conducting the guided inquiry, making these approaches suitable for more senior learners.

After aligning the UBC model and interprofessional socialisation framework of IPE, the results of the systematic review indicate that the IPE learning and teaching approaches applied in undergraduate curricula were mostly at the stage of awareness and exposure (uniprofessional identity), followed by immersion and application (interprofessional role learning) (Fig. 2). It is understandable that the approaches were mainly distributed between stage one and stage two of the IPE learning process and the interprofessional socialization framework because the focus of this review was on undergraduate IPE activities. Stage one of the IPE learning process is represented by PBL, TBL, self-directed learning, and other learning approaches. This stage works as a foundation for undergraduate junior students, in that it prepares them for the transformational learning that will take place in stages two and three by challenging their perceptions of their own profession and the roles played by other healthcare professionals [9]. In stage two, the senior undergraduate students collaborate with members of other professions and gain further IPE competencies through simulation-based learning, e-learning, evidence-based practice, and blended learning. The least applied stage in the included studies was the mastery stage of learning (dual identity development) represented by competency-based education. The third stage is the most advanced in terms of knowledge, skills, and the understanding of one’s own and other professions’ roles. Additionally, this stage aids in developing a sense of belonging in IPE teams [9]. However, despite this, only two programmes have implemented competency-based learning in their undergraduate curricula [30, 45].

The bulk of the systematic reviews of IPE in the literature focussed on the effectiveness of IPE, with few publications assessing its delivery. One review published by Khan and colleagues in 2001 discussed IPE tools and teaching strategies for students in the healthcare professions [63]. The authors concluded that simulation-based education was the most frequent approach used to deliver IPE [63]. This finding is in agreement with the results of the current review, as simulation-based education was used as a means to deliver IPE in eight studies [1, 46, 48–50, 55, 56, 58]. Our findings further support the report published by Herath et al. in 2017, who summarised the features of IPE programmes in developed and developing countries, including the teaching and learning approaches [18]. A clear variation in the teaching and learning approaches of IPE was observed in universities globally [18]. In addition, a combination of different teaching and learning approaches was applied to deliver IPE rather than a single learning approach. PBL, CBL and TBL were frequently used to deliver IPE. Moreover, simulation-based education was applied as a dominant learning approach in many universities [18].

This review has several limitations. The first was the limited availability of comprehensive literature addressing IPE as part of an undergraduate curriculum. To overcome this limitation, the research team broadened the literature search at the beginning and conducted extensive and focussed scanning to ensure the inclusion of all relevant articles. In addition, a complete description of the teaching and learning approaches was lacking in the original studies. The terms ‘strategy’, ‘approach’ and ‘theory’ overlapped in the included studies.

The current systematic review shed light on the teaching and learning approaches used in delivering IPE activities within undergraduate curricula for the healthcare professions. Subsequent reviews should focus on the most effective IPE teaching and learning approaches that can produce the best outcomes related to students’ knowledge, attitude and behaviour. The review also highlighted a need for more advanced studies that investigate how IPE activities lead to changes in organisational practice. In other words, educators should explore whether IPE activities are resulting in changes beyond the individual level (i.e. knowledge, attitude and skills). Further, the review highlighted that educators are focussed on designing undergraduate IPE activities at the awareness and exposure level of the UBC IPE model rather than advancing IPE learning activities at the immersion and application level. Such advancement might be necessary to prepare students for their future roles as competent team members within interprofessional teams.

## Conclusions

The current systematic review demonstrated that the teaching and learning approaches for IPE varied substantially between universities. Simulation-based education, e-learning and PBL were the most frequently used learning approaches to deliver IPE to undergraduate students. The evidence synthesised could support IPE curriculum planners and educators when planning an IPE programme. The review also revealed a lack of IPE programmes in the Middle East region. More global IPE initiatives are required to meet the global healthcare workforce needs. Further studies are required to identify the effectiveness of the different learning approaches in the development of IPE competencies.

## Supplementary Information

**Additional file 1.** Search strategy for identification of articles for the review of undergraduate-level teaching and learning approaches for interprofessional education in the healthcare professions.

**Additional file 2.** Data extraction tool that was designed and used to extract data from the articles included in the current systematic review.

**Additional file 3.** Original quality checklist for mixed-methodology case studies and other in-depth complex designs [42].

**Additional file 4.** Checklist used for assessing quality of included articles. The checklist was adapted from Mays et al. 2001 [42].

**Additional file 5.** Modified Kirkpatrick’s framework. The framework was adapted from Barr’s six-level classification [43, 44].

**Additional file 6.** Overview of the included studies that implemented interprofessional education (IPE) programs in undergraduate curricula.

## Data Availability

The datasets used during the current study are available from the corresponding author on reasonable request.

## Abbreviations

CBL: Case-Based Learning
TBL: Team-Based Learning
PBL: Problem-Based Learning
IPE: Interprofessional Education
WHO: World Health Organization
ELT: Experiential Learning Theory
PEO: Participants, Educational Aspects, and Outcomes Model
PRISMA: Preferred Reporting Items for Systematic Reviews and Meta-Analyses
UBC: University of British Columbia
MPE: Multiprofessional Education
