# Supplementary material for: Undergraduate-level teaching and learning approaches for interprofessional education in the health professions: a systematic review
Source: BMC Med Educ. 2022 Jan 3;22:13. doi: 10.1186/s12909-021-03073-0 (PMC8725543; doi:10.1186/s12909-021-03073-0)
Supplement: Supplementary file 1 — Additional file 1. Search strategy for identification of articles for the review of undergraduate-level teaching and learning approaches for interprofessional education in the healthcare professions. [file 12909_2021_3073_MOESM1_ESM.docx]

**Additional File 1: Search strategy for identification of articles for the review of undergraduate-level teaching and learning approaches for interprofessional education in the healthcare professions**

| **Keywords** | **PubMed** | **Science Direct** | **Cochrane Controlled Trial Register** |
| --- | --- | --- | --- |
| “*Interprofessional education*” OR “*multiprofessional education*” AND “*undergraduate*” | 832 | 316 | 193 |
| “*Interprofessional education*” OR “*multiprofessional education*” AND “*prelicensure*” | 59 | 309 | 243 |
| “*Interprofessional education*” OR “*multiprofessional education*” AND “*prequalification*” | 2 | 309 | 204 |
| “*Interprofessional learning*” OR “*multiprofessional learning*” AND “*undergraduate*” | 497 | 221 | 117 |
| “*Interprofessional learning*” OR “*multiprofessional learning*” AND “*prelicensure*” | 35 | 209 | 116 |
| “*Interprofessional learning*” OR “*multiprofessional learning*” AND “*prequalification*” | 1 | 209 | 115 |
| **Total** | **1426** | **1573** | **988** |
