# Supplementary material for: Undergraduate-level teaching and learning approaches for interprofessional education in the health professions: a systematic review
Source: BMC Med Educ. 2022 Jan 3;22:13. doi: 10.1186/s12909-021-03073-0 (PMC8725543; doi:10.1186/s12909-021-03073-0)
Supplement: Supplementary file 2 — Additional file 2. Data extraction tool that was designed and used to extract data from the articles included in the current systematic review. [file 12909_2021_3073_MOESM2_ESM.docx]

**Additional File 2: Data extraction tool that was designed and used to extract data from the articles included in the current systematic review**

|  |  |  | **IPE program** | | | | | | | | |
| --- | --- | --- | --- | --- | --- | --- | --- | --- | --- | --- | --- |
| **Citation and title of the article** | **Institution(s)** | **Country** | **Participating health professions** | **Settings and contexts** | **Learning and teaching approaches** | **Placement within curriculum** | **Integrated or independent within curriculum?** | **Duration** | **Elective or compulsory?** | **Evaluation method** | **Findings** |
|  |  |  |  |  |  |  |  |  |  |  |  |
|  |  |  |  |  |  |  |  |  |  |  |  |
|  |  |  |  |  |  |  |  |  |  |  |  |
|  |  |  |  |  |  |  |  |  |  |  |  |
|  |  |  |  |  |  |  |  |  |  |  |  |
|  |  |  |  |  |  |  |  |  |  |  |  |
|  |  |  |  |  |  |  |  |  |  |  |  |
|  |  |  |  |  |  |  |  |  |  |  |  |
|  |  |  |  |  |  |  |  |  |  |  |  |
|  |  |  |  |  |  |  |  |  |  |  |  |
|  |  |  |  |  |  |  |  |  |  |  |  |
|  |  |  |  |  |  |  |  |  |  |  |  |
|  |  |  |  |  |  |  |  |  |  |  |  |
