# Supplementary material for: Undergraduate-level teaching and learning approaches for interprofessional education in the health professions: a systematic review
Source: BMC Med Educ. 2022 Jan 3;22:13. doi: 10.1186/s12909-021-03073-0 (PMC8725543; doi:10.1186/s12909-021-03073-0)
Supplement: Supplementary file 4 — Additional file 4. Checklist used for assessing quality of included articles. The checklist was adapted from Mays et al. 2001 [42]. [file 12909_2021_3073_MOESM4_ESM.docx]

**Additional File 4: Checklist used for assessing quality of included articles**

The checklist was adapted from Mays et al. 2001 [42].

| Study title: | | | | |
| --- | --- | --- | --- | --- |
| Criteria | Yes | No | Unclear | Not applicable |
| 1. Question. Did the study/project address a specific question/aim? |  |  |  |  |
| 1. Design. Was the study design explained? |  |  |  |  |
| 1. Funding. Did the paper clarify if the study/project was funded? If yes, by whom? |  |  |  |  |
| 1. Resource system. Was the origin of the study/project stated? |  |  |  |  |
| 1. Innovation. Was the nature/background of the innovation illustrated? |  |  |  |  |
| 1. Context. Was the context of the study/project sufficiently described? |  |  |  |  |
| 1. User system. Were the participants in the study/project elucidated? |  |  |  |  |
| 1. Implementation mechanism. Was the implementation process explained? |  |  |  |  |
| 1. Sampling. Did the authors include sufficient cases/settings/observations so that conceptual rather than statistical generalisations could be made? |  |  |  |  |
| 1. Data collection. Was the data collection process systematic, thorough and auditable? |  |  |  |  |
| 1. Data analysis. Were data analysed systematically and rigorously? Were sufficient data presented? |  |  |  |  |
| 1. Results. Were the main results stated with enough details? |  |  |  |  |
| 1. Conclusions. Did the authors draw a clear link between data and explanation (theory)? |  |  |  |  |
| 1. Reflexivity. Were the authors’ positions and roles clearly explained and the resulting biases considered? |  |  |  |  |
| 1. Ethics. Was the declaration of interest stated? |  |  |  |  |
| Final decision: | | | | |
| Comment: | | | | |
| Reviewer: | | | | |
