# Supplementary material for: Undergraduate-level teaching and learning approaches for interprofessional education in the health professions: a systematic review
Source: BMC Med Educ. 2022 Jan 3;22:13. doi: 10.1186/s12909-021-03073-0 (PMC8725543; doi:10.1186/s12909-021-03073-0)
Supplement: Supplementary file 5 — Additional file 5. Modified Kirkpatrick’s framework. The framework was adapted from Barr’s six-level classification [43, 44]. [file 12909_2021_3073_MOESM5_ESM.docx]

**Additional File 5: Modified Kirkpatrick’s framework**

The framework was adapted from Barr’s six-level classification [43, 44].

| **Level** | **Classification** | **Description** |
| --- | --- | --- |
| Level 1 | Participant reaction | Perspectives of students about interprofessional education (IPE) experience and its nature |
| Level 2a | Change in attitudes | Identify changes in attitudes towards team members of the IPE groups |
| Level 2b | Change in knowledge and skills | Identify changes in knowledge and skills related to the IPE learning activity |
| Level 3 | Behavioural change | Identify individual transfer of IPE to changes in practice settings |
| Level 4a | Change in organisational practice | Identify broad changes in organisational practice and delivery of patient care |
| Level 4b | Change in clinical outcome | Identify improvements in patient care |
