# Supplementary material for: Undergraduate-level teaching and learning approaches for interprofessional education in the health professions: a systematic review
Source: BMC Med Educ. 2022 Jan 3;22:13. doi: 10.1186/s12909-021-03073-0 (PMC8725543; doi:10.1186/s12909-021-03073-0)
Supplement: Supplementary file 6 — Additional file 6. Overview of the included studies that implemented interprofessional education (IPE) programs in undergraduate curricula. [file 12909_2021_3073_MOESM6_ESM.docx]

**Additional File 6: Overview of the included studies that implemented interprofessional education (IPE) programs in undergraduate curricula**

|  |  |  | **IPE program** | | | | | | | | |
| --- | --- | --- | --- | --- | --- | --- | --- | --- | --- | --- | --- |
| **Citation and title of the article** | **Institution(s)** | **Country** | **Participating health professions** | **Settings and contexts** | **Learning and teaching approaches** | **Placement within curriculum** | **Integrated or independent within curriculum?** | **Duration** | **Elective or compulsory?** | **Evaluation method** | **Findings** |
| Van Lierop et al. (2019) [51]  ‘Jointly discussing care plans for real-life patients: The potential of a student-led interprofessional team meeting in undergraduate health professions education’. | Maastricht University  and  Zuyd University of Applied Sciences. | The Netherlands. | Medicine, physiotherapy, occupational therapy, speech and language therapy and nursing. | University-based.  Cases for real-life and vulnerable elderly patients. | Problem-based learning. | Maastricht University: part of students’ clinical rotation in family medicine and social medicine.  Zuyd University: IPE building blocks were formulated based on existing competency models. | Integrated. | Every four weeks. Consisted of six concurrent IPE teams, each containing nine to ten participants from different health professions.  Each IPE team meeting lasted 2.5 hours. | Compulsory. | Qualitative: focus groups. | The students reported positive experiences with the IPE program. |
| Rodríguez et al. (2019) [52]  ‘Developing creative and research skills through an open and interprofessional inquiry-based learning course’. | Pompeu Fabra University. | Spain. | Human biology and medicine. | University-based.  Research and creative thinking skills. | Open inquiry-based learning.  Creativity workshops. | A full and open interprofessional inquiry-based learning course, ‘Integrated Bio-medicine’, was offered to third-year students in the Bachelor of Human Biology and Bachelor of Medicine programs. | Integrated. | The course lasted ten weeks, with two-hour sessions each week. | Unclear. | Mixed method: surveys and focus groups. | The students were highly satisfied with the IPE learning experience, and they perceived it as useful for their education. |

**Additional File 6: Overview of the included studies that implemented interprofessional education (IPE) programs in undergraduate curricula** (*Continued*)

|  |  |  | **IPE program** | | | | | | | | |
| --- | --- | --- | --- | --- | --- | --- | --- | --- | --- | --- | --- |
| **Citation and title of the article** | **Institution(s)** | **Country** | **Participating health professions** | **Settings and contexts** | **Learning and teaching approaches** | **Placement within curriculum** | **Integrated or independent within curriculum?** | **Duration** | **Elective or compulsory?** | **Evaluation method** | **Findings** |
| Van Gessel et al. (2018) [1]  ‘Interprofessional training: Start with the youngest! A program for undergraduate healthcare students in Geneva, Switzerland’. | The University of Applied Sciences and its Healthcare School  and  The University of Geneva. | Switzerland. | Nutrition, physiotherapy, midwifery, nursing, medical radiology, dentistry, and medicine. | University-based.  Team Strategies and Tools to Enhance Performance and Patient Safety. | Simulation-based learning.  Interprofessional congress: plenary sessions and workshops. | Three sequential modules: totalised 300 h of teaching and learning for around 1400–1500 students from six tracks. | Integrated. | Module 1: a full week.  Module 2: two semesters.  Module 3: six months. | Compulsory. | Quantitative: surveys. | The students showed positive attitudes towards the implementation of the IPE program. |
| Imafuku et al. (2018) [53]  ‘What did first-year students experience during their interprofessional education? A qualitative analysis of e-portfolios’. | Showa University. | Japan. | Nursing, occupational therapy, physical therapy, medicine, dentistry, and pharmacy. | University-based, hospital-based, and community-based.  Problems related to nutrition balance, heatstroke or a cancer patient and family support. | Problem-based learning.  Early exposure.  Interactive lecture-based teaching.  Laboratory work. | The first-year IPE programme consisted of five modules. | Integrated. | 1 year. | Unclear. | Qualitative: written reflections from students’ e-portfolios. | The students’ active involvement in the IPE programme facilitated their understanding of communication/teamwork and identity formation as a health care professional in an interprofessional collaborative practice. |

**Additional File 6: Overview of the included studies that implemented interprofessional education (IPE) programs in undergraduate curricula** (*Continued*)

|  |  |  | **IPE program** | | | | | | | | |
| --- | --- | --- | --- | --- | --- | --- | --- | --- | --- | --- | --- |
| **Citation and title of the article** | **Institution(s)** | **Country** | **Participating health professions** | **Settings and contexts** | **Learning and teaching approaches** | **Placement within curriculum** | **Integrated or independent within curriculum?** | **Duration** | **Elective or compulsory?** | **Evaluation method** | **Findings** |
| Milot et al. (2015) [54]  ‘Building an interfaculty interprofessional education curriculum: What can we learn from the Université Laval Experience?’. | Université Laval. | Canada. | Occupational therapy, physiotherapy, social work, kinesiology, medicine, nursing, nutrition, and pharmacy. | University-based.  The Canadian interprofessional health collaborative. | Blended learning combined e-learning (synchronous) and face-to-face activities. | An undergraduate IPE curriculum of three courses, configured and scheduled in a consecutive and sequential manner.  Three one-credit courses. | Integrated. | Curriculum courses were offered on the timetable every semester (fall, winter, summer) as a part of a 45-h program that was divided into three courses. | Compulsory. | Not applicable. | Not applicable. |
| Sanborn H. (2016) [30]  ‘Developing asynchronous online interprofessional education’. | Arizona State University College of Nursing and Health Innovation. | USA. | Author didn't mention participated health professions. | University‐based.  Interprofessional education collaborative competencies. | Competency-based learning.  Experiential learning.  Discussion boards.  Written papers.  Presentations.  Virtual games (asynchronous). | The IPE project resulted in 61 new IPE objectives, integrated through ten courses. | Integrated. | Author didn't mention duration. | Unclear. | Not applicable. | Not applicable. |

**Additional File 6: Overview of the included studies that implemented interprofessional education (IPE) programs in undergraduate curricula** (*Continued*)

|  |  |  | **IPE program** | | | | | | | | |
| --- | --- | --- | --- | --- | --- | --- | --- | --- | --- | --- | --- |
| **Citation and title of the article** | **Institution(s)** | **Country** | **Participating health professions** | **Settings and contexts** | **Learning and teaching approaches** | **Placement within curriculum** | **Integrated or independent within curriculum?** | **Duration** | **Elective or compulsory?** | **Evaluation method** | **Findings** |
| Horsley et al. (2016) [55]  ‘Developing a foundation for interprofessional education within nursing and medical curricula’. | Loyola University Chicago. | USA. | Nursing and medicine. | University-based.  Team Strategies and Tools to Enhance Performance and Patient Safety. | Simulation-based learning. | Nursing: within the last one to two semesters of the nursing program  Medicine: in the fourth year during emergency medicine clerkship. | Nursing: independent.  Medicine: integrated. | IPE simulations are conducted once a month for 11 months of the calendar year. | Compulsory. | Not applicable. | Not applicable. |
| Meche et al. (2015) [56]  ‘Design and implementation of an interprofessional education course for undergraduate students at the University of Applied Sciences Western Switzerland: the Geneva experience’. | University of Applied Sciences Western Switzerland. | Switzerland. | Nursing, physiotherapy, nutrition, midwifery, and medical radiology. | University-based.  Emergency situations and multicultural contexts. | Simulation-based learning.  Scenario-based learning.  Discussion of fictional clinical cases.  Health promotion and prevention projects.  E-learning**. | Ten ECTS* of the 180 Swiss Bachelor of Health credits are validated with IPE. | Unclear. | Yearly basis. | Unclear. | Not applicable. | Not applicable. |

**^*^** ECTS: European Credit Transfer and Accumulation System

^**^ Unclear whether e-learning approach was synchronous or asynchronous

**Additional File 6: Overview of the included studies that implemented interprofessional education (IPE) programs in undergraduate curricula** (*Continued*)

|  |  |  | **IPE program** | | | | | | | | |
| --- | --- | --- | --- | --- | --- | --- | --- | --- | --- | --- | --- |
| **Citation and title of the article** | **Institution(s)** | **Country** | **Participating health professions** | **Settings and contexts** | **Learning and teaching approaches** | **Placement within curriculum** | **Integrated or independent within curriculum?** | **Duration** | **Elective or compulsory?** | **Evaluation method** | **Findings** |
| Waggie M. and Laattoe N. (2014) [57]  ‘Interprofessional exemplars for health professional programmes at a South African university’. | University of the Western Cape. | South Africa. | Physiotherapy, occupational therapy, nursing, social work, nutrition, medicine, sport science and recreation. | University-based and community-based. | Small group discussions.  Didactic input.  Group work.  Role plays.  Presentations.  Reflections.  Case studies.  Video clips. | The IPE curriculum is offered at the first-, second- and third-year levels through the delivery of core interdisciplinary courses. | Unclear. | Unclear. | Unclear. | Not applicable. | Not applicable. |
| Hinderer K. and Joyner R. (2014) [58]  ‘An interprofessional approach to undergraduate critical care education’. | Salisbury University. | USA. | Nursing and respiratory care. | University‐based.  Critical care clinical practice. | Simulation-based learning.  Case studies.  Clinical observation.  Student patient case presentations. | The three-credit course was designed to fit within a traditional 15-week semester. | Unclear. | Offered one night per week in a 150-minute session. | Elective. | Quantitative: surveys. | The students reported an increased understanding of the importance of interprofessional collaboration. |

**Additional File 6: Overview of the included studies that implemented interprofessional education (IPE) programs in undergraduate curricula** (*Continued*)

|  |  |  | **IPE program** | | | | | | | | |
| --- | --- | --- | --- | --- | --- | --- | --- | --- | --- | --- | --- |
| **Citation and title of the article** | **Institution(s)** | **Country** | **Participating health professions** | **Settings and contexts** | **Learning and teaching approaches** | **Placement within curriculum** | **Integrated or independent within curriculum?** | **Duration** | **Elective or compulsory?** | **Evaluation method** | **Findings** |
| Vanier et al. (2013) [45]  ‘Innovating in teaching collaborative practice with a large student cohort at Université de Montréal’. | Université de Montréal. | Canada. | Audiology, medicine, nursing, nutrition, occupational therapy, pharmacy, physiotherapy, psychology, social work, and speech therapy. | University-based. | Competency-based learning.  Individual reading and assignments.  Small group discussions.  Videos. | Three one-credit courses. | Unclear. | Offered over the first three years of training. | Compulsory for all except psychology. | Quantitative: surveys. | The students demonstrated a positive impact on their understanding of collaborative practice and partnership in care. |
| Holland et al. (2013) [46]  ‘Interprofessional working in acute care’. | King’s College London. | UK. | Medicine, nursing and physiotherapy. | Hospital-based.  Acute care. | Simulation-based learning. Self-directed learning. In-practice teaching. Clinical observations. Reflections. Collaborative peer-group working. Students’ presentations. Blended learning included synchronous e-learning. Asynchronous discussions facilitated by a website. | 15-credit module. | Integrated. | Unclear. | Elective. | Qualitative: narrative feedback. | The student evaluations showed high satisfaction scores. |

**Additional File 6: Overview of the included studies that implemented interprofessional education (IPE) programs in undergraduate curricula** (*Continued*)

|  |  |  | **IPE program** | | | | | | | | |
| --- | --- | --- | --- | --- | --- | --- | --- | --- | --- | --- | --- |
| **Citation and title of the article** | **Institution(s)** | **Country** | **Participating health professions** | **Settings and contexts** | **Learning and teaching approaches** | **Placement within curriculum** | **Integrated or independent within curriculum?** | **Duration** | **Elective or compulsory?** | **Evaluation method** | **Findings** |
| Doucet et al. (2013) [47]  ‘A team approach to an undergraduate interprofessional communication course’. | University of New Brunswick. | Canada. | Nuclear medicine, nursing, radiation therapy, radiological technology and respiratory therapy. | University-based. | Team-based learning.  Face-to-face meetings.  Online discussion boards (synchronous). | One IPE course incorporated interprofessional competencies into the weekly learning activities. | Unclear. | Unclear. | Compulsory. | Mixed: readiness for interprofessional learning scale (RIPLS) and dear successor letters. | The students and course facilitators presented a positive experience of the IPE course. RIPLS demonstrated a trend towards improved interprofessional awareness. The ‘Dear Successor’ letters revealed a relatively high level of satisfaction with the course delivery. |
| Pardue K. (2013) [48]  ‘Not left to chance: Introducing an undergraduate interprofessional education curriculum’. | University of New England. | USA. | Applied exercise science, athletic training, dental hygiene, nursing and occupational therapy. | University-based. | Simulation-based learning. Problem-based learning.  Case-based learning.  Evidence-based practice. Role plays. | Two courses, totaling three credits, comprised the freshmen year, and two three-credit courses constituted the sophomore year. | Unclear. | Over 2 years. | Compulsory. | Not applicable. | Not applicable. |

**Additional File 6: Overview of the included studies that implemented interprofessional education (IPE) programs in undergraduate curricula** (*Continued*)

|  |  |  | **IPE program** | | | | | | | | |
| --- | --- | --- | --- | --- | --- | --- | --- | --- | --- | --- | --- |
| **Citation and title of the article** | **Institution(s)** | **Country** | **Participating health professions** | **Settings and contexts** | **Learning and teaching approaches** | **Placement within curriculum** | **Integrated or independent within curriculum?** | **Duration** | **Elective or compulsory?** | **Evaluation method** | **Findings** |
| Olenick et al. (2011) [49]  ‘A regional model of interprofessional education’. | The Commonwealth Medical College. | USA. | Nursing, occupational therapy, physical therapy, physician assistant, pharmacy, speech language pathology, nutrition, social work, health education, emergency medical services, dental hygiene, surgical and vascular technology, diagnostic medical sonography, echocardiography, phlebotomy, respiratory therapy, radiation technology, medical imaging, biomedical engineering, counselling and human services, and exercise science. | Community-based.  Patient-centred. | Simulation-based learning.  Collaborative care seminars.  Live web-based seminars (synchronous).  Virtual environment interactive exercises (synchronous).  Interprofessional panel discussions.  Case-based interprofessional sessions.  Evidence-based. | In the fourth year of the Commonwealth Medical College curriculum. | Integrated. | Unclear. | Unclear. | Not applicable. | Not applicable. |

**Additional File 6: Overview of the included studies that implemented interprofessional education (IPE) programs in undergraduate curricula** (*Continued*)

|  |  |  | **IPE program** | | | | | | | | |
| --- | --- | --- | --- | --- | --- | --- | --- | --- | --- | --- | --- |
| **Citation and title of the article** | **Institution(s)** | **Country** | **Participating health professions** | **Settings and contexts** | **Learning and teaching approaches** | **Placement within curriculum** | **Integrated or independent within curriculum?** | **Duration** | **Elective or compulsory?** | **Evaluation method** | **Findings** |
| Bilodeau et al. (2010) [50]  ‘Interprofessional education at Laval University: Building an integrated curriculum for patient-centred practice’. | The Laval University. | Canada. | Nursing, medicine, pharmacy, kinesiology, nutrition, physiotherapy, occupational therapy, and social work. | University-based and hospital-based. | Simulation-based learning.  Lectures.  Small group discussions.  Web-based discussion forums (synchronous).  Workshops.  Seminars.  Student patient case presentations. | **Component I:** A 45-h undergraduate curriculum was developed and offered as three 15-h courses.  **Component II:** The practical training in primary care clinical settings consisted of three experimental phases: Phase 1: four half-day educational workshops; Phase 2: one half-day seminar; Phase 3: a six-week period. | Integrated. | Each course was offered every academic semester.  The IPE classes were held during three non-consecutive weekends. | **Component I:** compulsory in nursing.  **Component II:** compulsory for family medicine residents. | Mixed: semi-structured interviews and questionnaires. | A majority of the students believed that the IPE course should be offered in their program and that it should be mandatory. |
